# Detection of Parkinson disease using multiclass machine learning approach

**DOI:** 10.1038/s41598-024-64004-9

**Published:** 2024-06-15

**Authors:** Saravanan Srinivasan, Parthasarathy Ramadass, Sandeep Kumar Mathivanan, Karthikeyan Panneer Selvam, Basu Dev Shivahare, Mohd Asif Shah

**Affiliations:** 1 Department of Computer Science and Engineering, Vel Tech Rangarajan Dr. Sagunthala R&D Institute of Science and Technology, Chennai, 600062 India; 2 School of Computer Science and Engineering, Galgotias University, Greater Noida, 203201 India; 3 Department of Computer Applications, School of Computer Science Engineering and Information Systems, Vellore Institute of Technology, Vellore, Tamil Nadu 632014 India; 4 Department of Economics, Kabridahar University, Po Box 250, Kebri Dehar, Ethiopia; 5 Division of Research and Development, Lovely Professional University, Phagwara, Punjab 144001 India

**Keywords:** Machine learning, Feed-forward neural network, RandomizedSearchCV, SMOTE, Voice signal feature, Cancer, Diseases, Health care, Medical research, Risk factors

## Abstract

Parkinson’s Disease (PD) is a prevalent neurological condition characterized by motor and cognitive impairments, typically manifesting around the age of 50 and presenting symptoms such as gait difficulties and speech impairments. Although a cure remains elusive, symptom management through medication is possible. Timely detection is pivotal for effective disease management. In this study, we leverage Machine Learning (ML) and Deep Learning (DL) techniques, specifically K-Nearest Neighbor (KNN) and Feed-forward Neural Network (FNN) models, to differentiate between individuals with PD and healthy individuals based on voice signal characteristics. Our dataset, sourced from the University of California at Irvine (UCI), comprises 195 voice recordings collected from 31 patients. To optimize model performance, we employ various strategies including Synthetic Minority Over-sampling Technique (SMOTE) for addressing class imbalance, Feature Selection to identify the most relevant features, and hyperparameter tuning using RandomizedSearchCV. Our experimentation reveals that the FNN and KSVM models, trained on an 80–20 split of the dataset for training and testing respectively, yield the most promising results. The FNN model achieves an impressive overall accuracy of 99.11%, with 98.78% recall, 99.96% precision, and a 99.23% f1-score. Similarly, the KSVM model demonstrates strong performance with an overall accuracy of 95.89%, recall of 96.88%, precision of 98.71%, and an f1-score of 97.62%. Overall, our study showcases the efficacy of ML and DL techniques in accurately identifying PD from voice signals, underscoring the potential for these approaches to contribute significantly to early diagnosis and intervention strategies for Parkinson’s Disease.

## Introduction

Parkinson's Disease (PD) exacts a significant global toll, affecting millions and unfolding as a progressively debilitating condition characterized by the gradual emergence of symptoms. While overt signs commonly appear in individuals aged 50 and above, a surprising one in ten people experiences PD symptoms even before reaching the age of 40^1^. The core pathology of PD lies in the degeneration of specific nerve cells within the substantia nigra of the brain. These cells, responsible for the production of dopamine, a neurotransmitter critical for regulating bodily movements, face demise, leading to a deficiency of this vital chemical. The insufficiency of dopamine sets the stage for the gradual onset of a myriad of progressive symptoms associated with PD^2^. Initial manifestations often include tremors or stiffness localized to one side of the body, frequently affecting the hand or arm. As the disease progresses, it can culminate in the development of potential dementia in later stages, adding another layer of complexity to the challenges faced by individuals with PD^3^. The two decades spanning from 1996 to 2016 witnessed a staggering surge in the global prevalence of PD, catapulting from 2.5 million to an alarming 6.1 million cases. This more than fourfold increase is intricately linked to the extension of life expectancy, contributing to a world with a burgeoning aging population^4^. The brain, functioning as the paramount control centre of the body, orchestrates a sophisticated interplay with various bodily functions. PD introduces a spectrum of debilitating symptoms, encompassing the partial or complete loss of motor reflexes, speech issues that can progress to eventual failure, atypical behavioural patterns, cognitive decline, and the erosion of other critical skills^5^. However, discerning between the natural cognitive declines associated with aging and the early symptoms of PD poses a formidable challenge. The overlapping nature of these indicators underscores the intricate diagnostic process required for timely and accurate identification, emphasizing the pressing need for heightened awareness and comprehensive research to advance our understanding of Parkinson's Disease^6^. In the year 2017, the prospective economic impact of Parkinson's Disease (PD) in the United States was estimated at a substantial $51.9 billion. This comprehensive assessment included indirect costs amounting to $14.2 billion, non-medical expenses totalling $7.5 billion, and an additional $4.8 billion allocated to disability income specifically linked to public works^7^. Given that a significant portion of individuals diagnosed with PD are aged 65 and above, projections indicate that the overall economic burden is poised to surge to nearly $79 billion by the year 2037^8^. The current diagnostic landscape for PD, outlined by the National Collaborating Centre for Chronic Conditions in 2006, heavily relies on invasive techniques, empirical testing, and thorough examinations^9^. Unfortunately, these conventional methods not only incur exorbitant costs but also exhibit inefficiency, necessitating complex equipment that often yields suboptimal accuracy. In light of these challenges, there is a pressing need for innovative diagnostic approaches that prioritize cost-effectiveness, simplicity, and reliability in the diagnosis and treatment of PD. While non-invasive diagnostic techniques for PD remain relatively unexplored, the emerging field of machine learning presents promising avenues for advancement^10^. Machine learning techniques, by efficiently classifying individuals with PD and those without, leverage distinctive vocal patterns associated with the disorder for early and accurate detection. In pursuit of these objectives, this study aims to employ cutting-edge Machine Learning (ML) and Deep Learning (DL) models to differentiate between healthy individuals and those afflicted by PD based on distinctive voice signal features^11^.

Beyond the quest for enhanced diagnostic precision, the overarching goal of this research is to potentially alleviate some of the economic burdens associated with the comprehensive management of Parkinson's Disease. Through innovative, non-invasive methods, the study aspires to contribute to a more efficient and cost-effective paradigm for diagnosing and addressing the complexities of PD in the future^12^. Numerous investigations in neuroimaging have explored various non-invasive brain imaging techniques, including functional MRI (fMRI) and diffusion MRI (dMRI), in an attempt to tackle the challenges posed by autism spectrum disorder (ASD)^13^. While these studies have significantly advanced our comprehension of functional and structural connectivity changes in the brains of individuals with ASD, they have largely overlooked the intricate morphological alterations occurring between different brain regions^14^. Recognizing this gap within the field of network neuroscience, recent research endeavours have delved into the potential of cortical morphological networks (CMNs), exclusively derived from T1-weighted MRI, as a means to differentiate between the cortices of individuals with autism and those considered typical^15^. Notably, prior works have scrutinized changes in morphology at the level of individual brain regions. However, these studies have fallen short in investigating the dynamic alterations in one specific region of interest (ROI) concerning another ROI. In contrast, the morphological relationships between pairs of ROIs can be effectively modelled using morphological brain networks, where the connectivity between two regions encapsulates their dissimilarity in morphology, a concept recently introduced to the field^16^. This approach offers a nuanced understanding of the intricate morphological changes occurring at the interplay of specific brain regions in individuals with ASD, addressing a crucial aspect that previous studies had overlooked. Hence, this research endeavour seeks to detect Parkinson’s disease (PD) by employing advanced Machine Learning (ML) and Deep Learning (DL) models. The primary objective is to distinguish between individuals who are healthy and those with PD, leveraging distinctive features extracted from voice signals. The potential outcome aims not only to enhance diagnostic precision but also to contribute to the reduction of associated financial costs. The article is organized into distinct chapters. In Chapter 2, we explore various methods sourced from previously published articles. Moving on to Chapter 3, we delve into the utilization of datasets and the formulation of our proposed model architecture. Chapter 4 is dedicated to the discussion of the experimental results and the ensuing analysis. Finally, Chapter 5 addresses the conclusion of the study and outlines areas for future work.

## Related work

A multitude of researchers has actively engaged in the complex task of categorizing Parkinson's disease, employing a diverse array of methodologies. This collective effort has laid a robust foundation for the effective application of machine learning in tackling the inherent challenges associated with Parkinson's disease, particularly in the realms of subclassification, risk assessment, and prognosis, utilizing distinctive voice signal features. The adopted diagnostic technique integrates a seamless fusion of selection and classification procedures, incorporating sophisticated methodologies such as Feature Importance and Recursive Feature Elimination in the pivotal task of feature selection. In the course of extensive trials, a comprehensive spectrum of machine learning models has been leveraged, encompassing the versatile capabilities of artificial neural networks, support vector machines, and classification and regression trees. These models collectively served as instrumental tools in the intricate process of categorizing individuals with Parkinson's disease. Upon meticulous evaluation of the performance of these machine learning techniques, it became evident that the Support Vector Machines, particularly when coupled with Recursive Feature Elimination, exhibited superior outcomes. This innovative approach yielded a remarkable accuracy rate of 93.84%, achieved with the utilization of the minimal set of vocal features essential for Parkinson's diagnosis^17^.

The notable efficacy demonstrated by this methodology underscores its potential significance within the domain of neurodegenerative disease classification, offering a promising avenue for the refinement and enhancement of diagnostic approaches in the pursuit of improved patient outcomes. The findings stemming from the utilization of artificial neural networks and support vector machines as diagnostic tools for specialists handling Parkinson's disease are marked by a noteworthy accuracy level, standing at an impressive approximately 90%. These advanced computational models contribute significantly to the diagnostic process, demonstrating their effectiveness in assisting healthcare professionals in accurately identifying and evaluating Parkinson's disease cases^18^. This high level of accuracy not only underscores the reliability of these computational methods but also holds promising implications for enhancing the precision and efficiency of diagnostic procedures in the realm of Parkinson's disease diagnosis. As we delve into the details of these outcomes, it becomes evident that leveraging artificial neural networks and support vector machines proves to be a valuable asset in the ongoing efforts to improve the diagnostic landscape and ultimately enhance patient care within the domain of Parkinson's disease^19^. In the pursuit of accurate Parkinson's disease diagnosis and the efficient identification of healthy individuals, a comprehensive exploration of various classification techniques was undertaken. This study sought to discern the optimal approach for precision in distinguishing between affected and unaffected individuals. In the process, a meticulous comparative analysis unfolded, systematically employing four distinct classification schemes in a predefined sequence: Decision Trees, Regression, Neural Networks, and DM neural. Each classification scheme underwent thorough evaluation using a diverse set of assessment techniques, enabling a comprehensive understanding of their respective performances. Notably, among these classifiers, the neural network stood out as the most effective, substantiated by its consistently superior application scores across various evaluation metrics. The neural network's prowess in capturing nuanced patterns and intricate relationships within the dataset became evident, positioning it as the standout performer in the diagnostic context. This standout performance was further emphasized by the neural network's remarkable overall classification accuracy, reaching an impressive 92.9%^20^. This outcome underscores the efficacy of the neural network in accurately discerning Parkinson's disease cases and highlights its potential as a reliable tool for healthcare professionals in the diagnostic process. The findings contribute valuable insights into refining and optimizing classification techniques, paving the way for enhanced diagnostic precision and ultimately improving patient outcomes in the context of Parkinson's disease identification^21^. Author implemented a successful approach in Parkinson's disease identification by utilizing a deep belief network (DBN). This specific DBN configuration, tailored for voice template matching, incorporates an input mechanism derived from a feature extraction procedure. The DBN architecture employed in this study comprises two stacked Restricted Boltzmann Machines (RBMs) and one output layer, strategically harnessed for the purpose of categorizing Parkinson's disease. To optimize the network's parameters, a dual-stage learning process is implemented. The initial stage involves unsupervised learning, leveraging RBMs to mitigate potential challenges arising from unpredictable initial weights^22^.

Subsequently, the fine-tuning phase employs the backpropagation technique as a supervised learning approach, refining the network's performance. Experimental results are meticulously compared with diverse strategies and existing literature to underscore the effectiveness of the proposed system. Notably, the suggested approach surpasses all alternative methods, demonstrating an impressive total testing accuracy of 94%^23^. This outcome not only validates the efficacy of the proposed methodology but also positions it as a leading solution in the realm of Parkinson's disease identification, showcasing its potential for robust and accurate diagnostic applications^24^. The research proposed and evaluated two distinct classification schemes aimed at enhancing the accuracy of Parkinson's disease (PD) case identification through voice measurements. Initially, an adaptive moment-based backpropagation algorithm was applied to BPVAM, an artificial neural network. Subsequently, the researchers explored the integration of dimensionality reduction methods, specifically principal component analysis (PCA), in conjunction with BPVAM to classify the same dataset. The overarching objective was to elevate PD prediction accuracy in the early stages by augmenting the system's sensitivity to intricate data patterns. The outcomes revealed that the most favourable results were achieved by BPVAM and BPVAM PCA, exhibiting a noteworthy accuracy of 97.50%^25^. Following closely, the artificial neural network (ANN) with Levenberg–Marquardt demonstrated substantial accuracy at 95.89%. These findings underscore the effectiveness of the proposed classification schemes in refining PD case identification accuracy, highlighting their potential significance in early-stage prediction and diagnosis^26^. Cell categorization is a challenging task due to the presence of multiple cell categories. Current techniques mainly focus on tumor cells, but they struggle to accurately classify sick or normal cells. This research aimed to assess the effectiveness of classical machine learning and deep learning in categorizing normal and sick cells based on their characteristics. The study used machine learning techniques like logistic regression, support vector machine, and CNN to assess the accuracy of cell detection networks and reduce false-positive cells. The proposed technique demonstrated superior classification of normal and sick cells, with a CNN achieving a remarkable accuracy rate of 98% in identifying diseased cells. This breakthrough will enhance the practicality of using human sick cells in therapeutic settings^27^. The DNCIC framework, based on deep learning, can accurately predict drug-affected cells and normal mitochondria. It uses a convolutional neural network trained on confocal microscope images of mitochondria. The model has been verified using images of healthy and diseased cells. The proposed model has an accuracy of 98% in classifying two-photon excited fluorescence probe images, laying the groundwork for the diagnosis of drug-affected cells in evolutionary biology and precision medicine^28^. Author presented a convolutional neural network-based deep learning method called mitochondrial organelle movement classification (MOMC) for identifying mitochondrial movement. The method involves three steps: extracting local descriptions via GoogleNet, generating mid-level features using ResNet-50, and classifying the position of mitochondrial organelle movement using the Inception-V3 model. The approach includes a deep classification network, MOMC, and a verification network for accurate classification. The study found that CNNs classified mitochondrial organelle morphology more accurately than SVM, CNNs, and logistic regression. The convolutional neural network accurately detected mitochondrial movement with an accuracy of 96.32%^29^. EEG-based biometrics have limitations, such as noise-prone signals and multi-training/multi-channel acquisition. Stady-state visually evoked potential (SSVEP) offers advantages like high signal-to-noise ratio and untrained usage. However, few studies compare SSVEP to single-channel single-trial dry electrode-implemented biometric approaches using Recurrent Neural Networks (RNN). A promising single-channel SSVEP-based biometric approach using RNN deep models offers low-cost, user-friendly, and reliable individual identification authentication, potentially leading to significant application domains^30^. To determine hospitalization of patients exposed to arbovirus infections using machine learning algorithms on SISA and SISAL datasets. The results show improved accuracy and area under the curve scores (0.973) compared to previous research. The study also investigates gender recognition in arboviral infection medical records using recurrent neural network-based deep models, potentially influencing vector control policies to control the spread of arboviral infection^31^. Author proposed a machine learning algorithm-based prediction model for hospital patients and outpatients with suspected arboviral infection in urban areas. The model uses the Severity Index for Suspected Arbovirus (SISA) and SISAL datasets. The model achieves 100% accuracy and 1 AUC score, with 98.73% accuracy for the SISA dataset. The deep learning-based models show improved performance compared to previous studies, highlighting the importance of machine learning in diagnosing and comparing classification performances in resource-limited settings^32^.

The proposed K-Nearest Neighbor (KNN) and Feed-forward Neural Network (FNN) models aim to improve Parkinson's disease (PD) classification based on voice signal characteristics by leveraging advanced optimization strategies. These include addressing class imbalance with the Synthetic Minority Over-sampling Technique (SMOTE), selecting the most relevant features, and fine-tuning hyperparameters using RandomizedSearchCV.

## Material and methods

### Material

This study leverages a publicly accessible dataset housed by the University of Oxford (UO) repository in collaboration with the National Canter for Voice. Originally designed for general voice disorder research, the dataset encompasses voice recordings from 31 individuals: 23 diagnosed with Parkinson's Disease (PD) and 8 active controls (AC). Within the PD group, 16 are male and 7 are female, while the AC group consists of 3 males and 5 females. The dataset comprises 195 voice recordings, each captured for 36 s in a sound-treated booth. With the aid of a calibrated microphone positioned 8 cm from the individual's mouth, recordings were obtained. Each recording is characterized by 24 biomedical voice measurements^33^. An average of six recordings were made per participant, with 22 individuals providing six recordings and 9 providing seven. The age of the PD participants ranged from 46 to 85 years (mean: 65.8, standard deviation: 9.8), with diagnoses ranging from 0 to 28 years. For clarity, the "status" column in the dataset identifies individuals with PD as "1" and healthy controls as "0". This distinction facilitates the analysis and differentiation between the two groups. (https://archive.ics.uci.edu/dataset/174/parkinsons). Table 1 illustrates the detailed description of voice measures in UCI dataset.

**Table 1:** UCI dataset detailed information from^30^.

Voice measure	Meaning
Name	ASCII name of subject and recording number (categorical variables)
MDVP:Fo(Hz)	Average vocal fundamental frequency (Numerical variables)
MDVP:Fhi(Hz)	Maximum vocal fundamental frequency (Numerical variables)
MDVP:Flo(Hz)	Minimum vocal fundamental frequency (Numerical variables)
MDVP:Jitter(%)	Several measures of variation in fundamental frequency. (Numerical variables)
MDVP:Jitter(Abs)	
MDVP: RAP	
Jitter:DDP	
MDVP:Shimmer	Several measures of variation in amplitude (Numerical variables)
MDVP:Shimmer(dB)	
Shimmer: APQ3	
Shimmer: APQ5	
MDVP: APQ	
Shimmer:DDA	

### Methods

**Figure 1:**
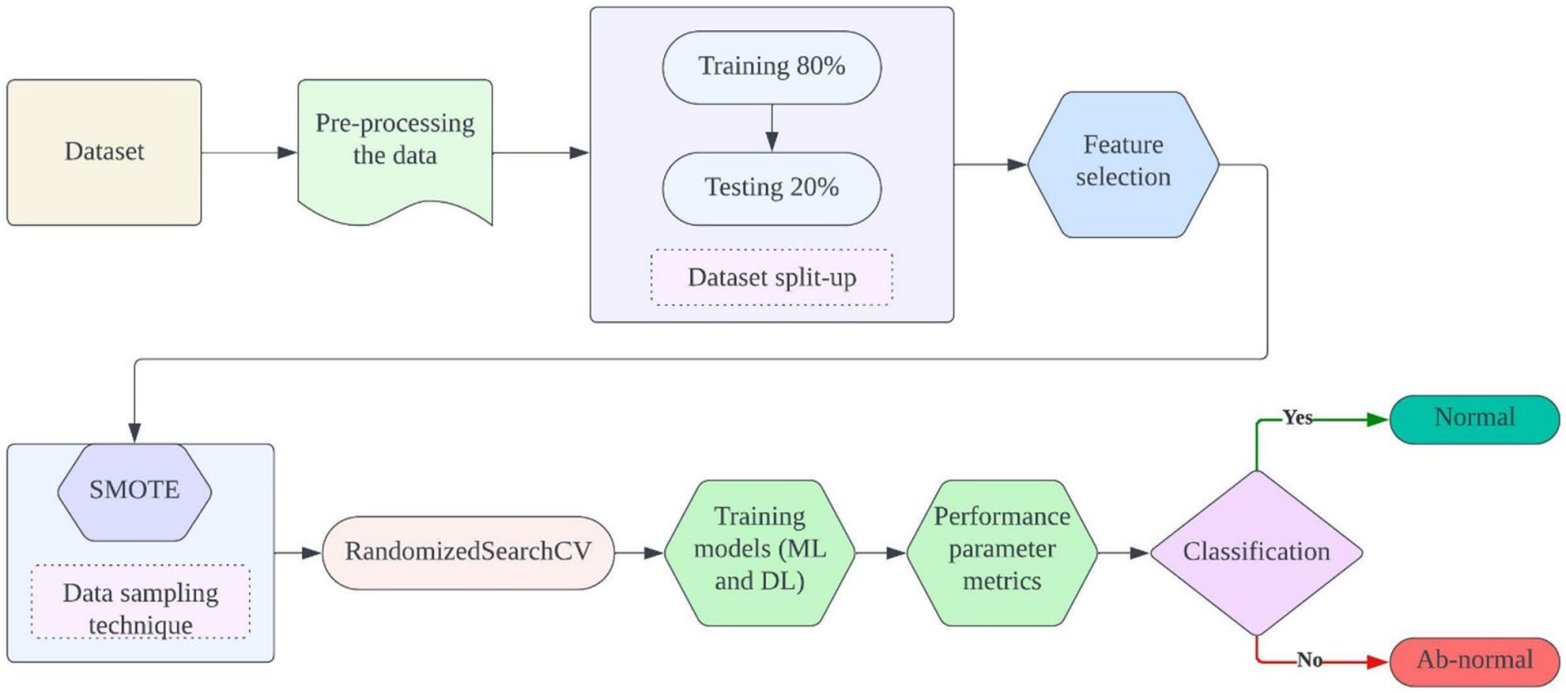
Proposed model architecture.

The proposed ensemble method for classifying Parkinson's Disease (PD) integrates various machine and deep learning models to enhance classification accuracy and robustness. Initially, meticulous data pre-processing ensures data quality and consistency, followed by the careful selection of relevant features to optimize model performance and mitigate overfitting. Addressing potential class imbalances, the Synthetic Minority Over-sampling Technique (SMOTE) strategically augments the minority class, ensuring a balanced dataset distribution. Subsequently, the RandomizedSearchCV algorithm systematically optimizes the hyperparameters of selected models, including K-Nearest Neighbor (KNN), Support Vector Machine (SVM), and Feed-forward Neural Network (FNN), maximizing predictive power. Evaluation metrics such as accuracy, precision, recall, and F1-score are rigorously employed to assess individual model performance. Through ensemble model construction, leveraging predictions from multiple models, the ensemble method capitalizes on the strengths of each constituent model to improve overall PD classification accuracy, offering a promising avenue for more effective patient diagnosis and treatment. Figure 1 depicts the proposed model architecture.

(i) *Data Collection*: The dataset used in this study consists of voice recordings from individuals with and without Parkinson's disease. These recordings have been converted into structured CSV format, capturing various vocal features such as pitch, jitter, shimmer, and harmonic-to-noise ratio.

(ii) *Preprocessing*: To address class imbalance in the dataset, we applied the Synthetic Minority Over-sampling Technique (SMOTE). This technique generates synthetic samples for the minority class, ensuring a balanced distribution of Parkinson's and healthy cases in the training set.

(iii) *Feature Selection*: We employed Recursive Feature Elimination (RFE) to identify the most relevant vocal features for Parkinson's disease classification. RFE iteratively removes the least important features based on the performance of a Support Vector Machine (SVM) model, ultimately selecting a subset of features that contribute most to the classification task.

(iv) *Model Development*: We developed two primary models: K-Nearest Neighbor (KNN) and Feed-forward Neural Network (FNN).

**Figure 2:**
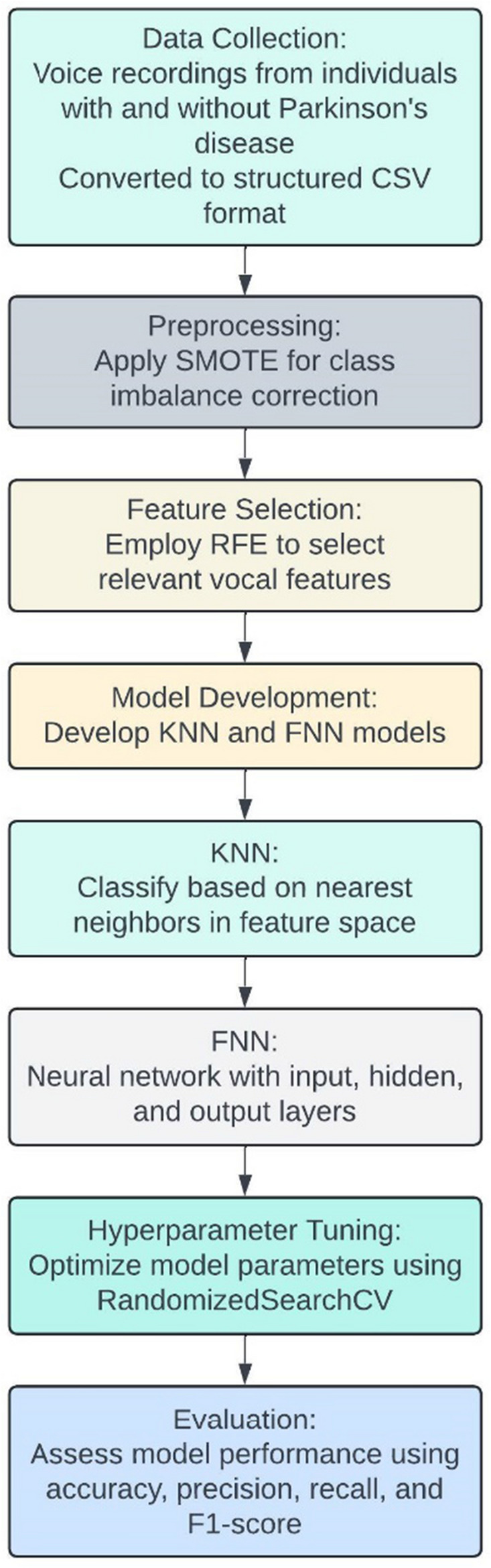
Operational flow diagram of proposed model.

(v) *KNN*: This model classifies individuals based on the majority class of their nearest neighbors in the feature space.

(vi) *FNN*: This neural network consists of an input layer, one or more hidden layers, and an output layer. We optimized the network architecture and parameters using RandomizedSearchCV.

(vii) *Hyperparameter Tuning*: For both models, we conducted hyperparameter tuning using RandomizedSearchCV, which randomly samples a wide range of parameter combinations to identify the optimal settings that maximize model performance.

(viii) *Evaluation*: The performance of the models was evaluated using accuracy, precision, recall, and F1-score. These metrics provide a comprehensive assessment of the models' ability to correctly identify individuals with and without Parkinson's disease. Figure 2 depicts operational flow diagram of the proposed model.

#### Pre-processing the Data

Preprocessing is a critical aspect of data processing that helps the model learn the features of the data effectively and remove unnecessary information^34^. Handling missing values is crucial to ensure the integrity of the dataset and the effectiveness of subsequent analyses. In our study, after importing the dataset into the Google Colab platform as a CSV file using the Pandas package, we conducted a thorough examination for duplicates and missing entries. Missing values can significantly impact model performance if not handled properly. To address this, we employed a combination of imputation strategies based on the nature and distribution of the missing data. For numerical features, we utilized mean imputation, where missing values were replaced with the mean value of the respective feature. This approach is effective when the missing data is deemed to be missing at random, and replacing it with the mean helps maintain the overall distribution of the data. Conversely, for categorical features, we applied mode imputation, replacing missing values with the most frequent category. This ensures that the imputed values are representative of the common trends in the dataset and helps preserve the integrity of the categorical variables. Following the imputation process, we observed that the dataset exhibited an imbalance, with 147 cases of Parkinson's disease (PD) and 48 healthy controls (HC), representing 75% and 25% of the dataset, respectively. To mitigate potential issues related to model performance, such as underfitting or overfitting, we partitioned the dataset into a 70:30 train/test split. This allowed us to train the model on a sufficient amount of data while reserving a portion for independent validation. Moreover, each feature was individually scaled using StandardScaler, a preprocessing technique that standardizes features by removing the mean and scaling to unit variance. This step ensures that all features contribute equally to the model and prevents features with larger magnitudes from dominating the learning process.

1 $$Standarization=\frac{a-\alpha }{\beta }$$

Here, α is referred as mean and β is referred as standard deviation. Seaborn and Matplotlib formed the cornerstone of data visualization in Python, allowing the creation of 2D graphs when coupled with libraries like Matplotlib, Pandas, and NumPy. Noteworthy in our toolkit was Scikit-learn, Python's formidable machine learning package. Its consistent interface, built on Python, provides tools for dimensionality reduction, grouping, regression, and classification. In essence, our approach encompassed a comprehensive data processing pipeline, leveraging diverse libraries to ensure effective model training and analysis.

#### Feature selection

In the critical phase of our data processing pipeline, we incorporated a cutting-edge dimensionality reduction technique called SelectKBest. This sophisticated algorithm played a pivotal role in optimizing our dataset by meticulously selecting the eight most informative features. Our strategic decision aimed to enhance the efficiency of subsequent modelling steps by focusing on the most relevant variables. SelectKBest, notable for being the second most widely adopted technique in dimensionality reduction, commands a substantial share of approximately 30% in overall usage. Its popularity underscores its effectiveness and widespread acceptance within the data science community. SelectKBest ranks features based on their "k score," a metric that gauges the relevance and informativeness of each feature to the target variable. The primary advantage of employing SelectKBest is its unparalleled capability to streamline and purify the dataset, ensuring that only the most relevant and influential features are retained for further analysis and model training. This strategic selection not only contributes to enhanced model interpretability but also significantly accelerates training times. The specific features selected by SelectKBest were determined based on their individual contribution to the predictive task at hand. Features that demonstrated the highest k scores were prioritized for inclusion in the final dataset, while less informative features were excluded. This process of feature selection was guided by the principle of maximizing predictive power while minimizing redundancy and overfitting. By prioritizing and retaining the most crucial information, SelectKBest acted as a catalyst in transforming our data into a more concise and efficient representation, laying a solid foundation for the subsequent stages of our data processing workflow.

#### SMOTE

SMOTE, which stands for Synthetic Minority Over-sampling Technique, is a powerful and widely used method in the realm of imbalanced machine learning datasets. Designed to address the challenge posed by uneven class distribution, SMOTE focuses on the minority class by generating synthetic instances, effectively balancing the representation of different classes in the dataset^35^. The core idea behind SMOTE is to alleviate the bias introduced by imbalanced datasets, where the minority class may be underrepresented. Rather than relying solely on the existing instances of the minority class, SMOTE creates synthetic examples by interpolating between the feature vectors of minority class instances. This is achieved by selecting a minority class instance and its nearest neighbours, and then generating new instances along the line segments connecting these neighbours. By introducing synthetic samples, SMOTE not only increases the number of minority class instances but also contributes to a more robust and balanced training set. This aids machine learning models in learning patterns from the minority class, preventing biases and improving overall predictive performance. It is particularly beneficial in scenarios where the minority class contains important and meaningful information that might be overshadowed by the dominance of the majority class. SMOTE has become a standard tool in the toolkit of data scientists and machine learning practitioners when dealing with imbalanced datasets. Its application helps mitigate the challenges associated with skewed class distributions, ultimately leading to more accurate and reliable models across various domains and applications.

1 $${z}_{j}{\prime}={z}_{j}+\nabla ({z}_{i}-{z}_{j})$$

2 $$SMOTE ({D}_{min},N, K)$$

Here, the components of the equation are defined as follows:

*D*_*min*_: Represents the minority class instances in the dataset that require over-sampling. *N*: Denotes the number of synthetic instances to be generated for each minority class instance. *k*: Specifies the number of nearest neighbours to be considered for each minority class instance during the synthetic sample generation process. The SMOTE equation captures the core elements of the algorithm, emphasizing the targeted over-sampling of minority class instances (D_min_) through the generation of synthetic instances. The parameters N and k play crucial roles in determining the quantity and characteristics of the synthetic instances introduced into the dataset. This technique is instrumental in addressing imbalances in class distribution, promoting a more equitable representation of minority and majority classes for improved machine learning model performance.

#### RandomizedSearchCV: hyper-parameter

RandomizedSearchCV, abbreviated for Randomized Search Cross-Validation, emerges as a robust strategy within the machine learning domain, dedicated to the streamlined refinement of hyperparameters. Departing from conventional grid search methodologies, which meticulously traverse the entire spectrum of hyperparameter combinations within a predefined search space, RandomizedSearchCV injects a dose of unpredictability into the process. This innovative approach involves the specification of hyperparameter distributions instead of fixed values. The optimization journey unfolds through the random sampling of hyperparameter values from these distributions over a predetermined number of iterations. This deliberate infusion of randomness empowers RandomizedSearchCV to traverse a varied landscape of hyperparameter combinations efficiently, presenting a computationally frugal alternative to exhaustive grid searches^36^.

Integrated seamlessly into the scikit-learn library, RandomizedSearchCV seamlessly aligns itself with machine learning models featuring adjustable hyperparameters. Leveraging the versatility of random sampling, this technique establishes itself as a potent and efficient conduit for hyperparameter tuning. Data scientists and machine learning practitioners find in RandomizedSearchCV a valuable ally, as it not only streamlines the hyperparameter tuning process but also enhances model performance and facilitates superior generalization on unseen data. The incorporation of randomness introduces an element of adaptability, allowing the algorithm to navigate the hyperparameter space dynamically, thus contributing to the iterative refinement of models in a more resource-conscious manner.

3 $$RandomizedSearchCV (M, P, scoring, cv, {n}_{iter})$$

**Figure 3:**
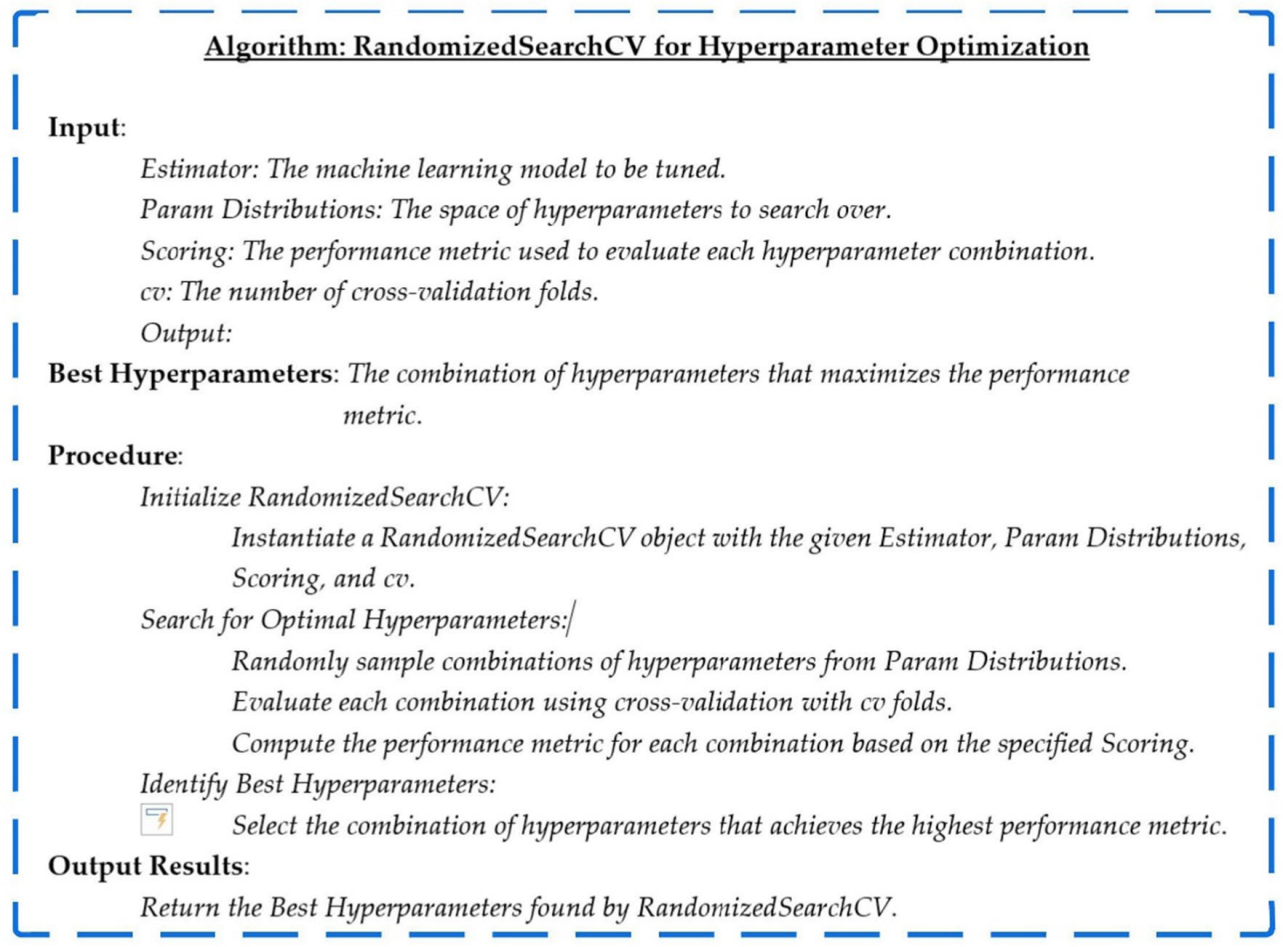
Algorithm for RandomizedSearchCV for hyperparameter optimization.

Here, the components of the equation are defined as follows:

*M*: Represents the machine learning model under consideration. This could be any scikit-learn compatible estimator, such as a classifier or a regressor. *P*: Denotes the hyperparameter space to be explored. Unlike grid search, which enumerates all possible combinations, defines probability distributions for each hyperparameter. *scoring*: Refers to the evaluation metric used to assess the performance of the model for each hyperparameter combination. Common choices include accuracy, precision, recall, or custom scoring functions. *cv*: Specifies the cross-validation strategy. This could be an integer (for k-fold cross-validation) or a cross-validation splitter object. *n_iter*: Represents the number of random combinations to sample from the hyperparameter space. This controls the balance between exploration and exploitation. The RandomizedSearchCV equation captures the dynamic exploration of hyperparameter space, introducing randomness to efficiently search for optimal model configurations while maintaining computational efficiency. Figure 3 depicts the RandomizedSearchCV algorithm.

#### Classification methods

In our study, we leverage a combination of Machine Learning (ML) and Deep Learning (DL) models to discern between individuals classified as healthy and those diagnosed with Parkinson's disease (PD). The diverse set of models includes the Kernel Support Vector Machine (KSVM), Random Forest (RF), Decision Tree (DT), K-Nearest Neighbor (KNN), and Feed-forward Neural Network (FNN). These models collectively operate on voice signal features, utilizing the distinctive patterns and characteristics embedded in the audio data to make accurate predictions about the health status of individuals. This ensemble of ML and DL models reflects a comprehensive approach to health classification based on voice signals. By harnessing the strengths of each model, we aim to create a sophisticated and accurate system capable of distinguishing between healthy individuals and those with Parkinson's disease, contributing to advancements in medical diagnosis and treatment.

##### Kernel Support Vector Machine

The Kernel Support Vector Machine (KSVM) is a powerful machine learning algorithm commonly used for classification and regression tasks. It belongs to the family of Support Vector Machines (SVMs) and is particularly effective when dealing with non-linearly separable datasets. The primary objective of a KSVM is to find a hyperplane in a high-dimensional space that best separates different classes of data. KSVM finds applications in various fields, including image classification, bioinformatics, and speech recognition, among others. Its ability to handle non-linear relationships in data and make accurate predictions even in high-dimensional spaces contributes to its popularity in the machine learning community. The formulation of the Kernel Support Vector Machine (KSVM) involves the optimization of a decision function that defines a hyperplane in a transformed feature space. The general equation for the decision function of a binary KSVM is expressed as follows:

4 $$f\left(x\right)=sign\left[{\sum }_{i=1}^{N}{\alpha }_{i}{y}_{i}K\left({x}_{i,}x\right)+b\right]$$

Here, the components of the equation are defined as follows:

$f\left(x\right)$ referred as decision function that classifies a new instance *x* based on the sign of the summation. *N*: The number of support vectors in the training dataset. ${\alpha }_{i}$ is represents the Lagrange multipliers associated with each support vector, determined during the optimization process. ${y}_{i}$ is the class label of the *i*^*th*^ support vector. The kernel function, evaluating the similarity between the *i*^*th*^ support vector *x*_*i*_ and the input instance *x* in the transformed space. *b* is the bias term, also known as the threshold, which shifts the decision boundary. The kernel function $K\left({x}_{i,}x\right)$ is a crucial element in the KSVM, determining the transformation applied to the input data. Common choices include the radial basis function (RBF) kernel and polynomial kernel, among others. The KSVM Eq. (4) illustrates how the decision function combines the contributions of support vectors, weighted by their Lagrange multipliers and class labels, to make predictions in the transformed feature space. This formulation allows KSVM to effectively handle non-linear relationships in the data.

##### Random forest

Random Forest is an ensemble learning algorithm that operates by constructing a multitude of decision trees during training and outputs the mode of the classes for classification tasks or the average prediction for regression tasks. The fundamental idea behind Random Forest is to introduce randomness in both the data and the features used for constructing the individual trees, thereby promoting diversity and improving the overall predictive performance. RF is widely used across various domains due to its flexibility, robustness, and ability to handle complex datasets. It is effective for both classification and regression tasks and is particularly popular in machine learning applications where interpretability, scalability, and high predictive accuracy are essential. The prediction equation for a Random Forest in the context of classification tasks is as follows:

5 $$\widehat{Y}=mode ({Y}_{1},{Y}_{2},{Y}_{3},\dots ,{Y}_{T})$$

Here, the components of the equation are defined as follows:

$\widehat{Y}$ is the predicted class for a new instance. ${Y}_{1},{Y}_{2},{Y}_{3},\dots ,{Y}_{T}$ are the individual predictions from each tree in the Random Forest. *T* is the total number of trees in the ensemble. In a RF, each tree is built independently through bootstrap sampling (sampling with replacement) from the training dataset. Additionally, at each split in each tree, only a random subset of features is considered, introducing randomness and decorrelating the trees. The final prediction is determined by a majority vote among the predictions of all the trees. In the case of regression tasks, the average of the predicted values from all trees is taken. RF algorithm excels in leveraging the collective wisdom of diverse decision trees, each trained on a different subset of the data and features. This ensemble approach leads to robust predictions, mitigating the risk of overfitting associated with individual trees and enhancing the model's generalization performance.

##### Decision tree

A Decision Tree is a supervised machine learning algorithm used for both classification and regression tasks. It operates by recursively partitioning the data into subsets based on the values of input features, ultimately assigning a class label or predicting a continuous value at each leaf node. The decision tree Eq. (6) captures the essence of the algorithm, emphasizing the recursive decision-making process based on feature values to partition the data and make predictions. Despite their simplicity, decision trees serve as building blocks for more complex ensemble methods, such as Random Forests and Gradient Boosting.

6 $$DT \left(X,Y\right)=Node (X,Y)$$

Here, the components of the equation are defined as follows:

DT (X, Y): The decision tree function that recursively constructs nodes based on the input features (X) and target labels (Y). Node (X, Y): A decision node in the tree, representing a split point based on the features. This decision node is constructed by selecting the best feature to split the data, and it leads to further recursive calls to the DT function for the subsets of data created by the split. The DT construction involves selecting features at each node to create decision points that partition the data into subsets. This process is repeated recursively until a stopping criterion is met, such as reaching a maximum depth, having a minimum number of samples in a node, or achieving perfect homogeneity.

##### K-nearest Neighbour

The KNN algorithm is a supervised machine learning algorithm used for both classification and regression tasks. It operates on the principle of proximity, making predictions for a new data point based on the majority class (for classification) or the average value (for regression) of its k-nearest neighbours in the feature space. The equation for the KNN algorithm can be summarized as follows:

7 $$\widehat{Y}=majority vote ({Y}_{1},{Y}_{2},{Y}_{3},\dots ,{Y}_{k})$$

$\widehat{Y}$ is the predicted class for a new instance. ${Y}_{1},{Y}_{2},{Y}_{3},\dots ,{Y}_{k}$ are the class labels or values of the k-nearest neighbours in the feature space. The choice of the distance metric and the value of k are crucial parameters in the KNN algorithm. Common distance metrics include Euclidean distance, Manhattan distance, and Minkowski distance. KNN is a simple and intuitive algorithm, but its performance can be sensitive to the choice of these parameters and the distribution of the data. It is a non-parametric and instance-based algorithm, meaning it does not make assumptions about the underlying data distribution and relies on the entire dataset for making predictions.

##### Feed-forward Neural Network

A Feed-forward Neural Network (FNN), also known as a multilayer perceptron (MLP), is a type of artificial neural network designed for supervised learning tasks, such as classification and regression. It consists of an input layer, one or more hidden layers, and an output layer. The term "feed-forward" refers to the flow of information through the network, where data travels from the input layer, passes through the hidden layers, and produces an output without forming cycles or loops. The mathematical representation of a feed-forward neural network involves a series of matrix operations, activation functions, and weight adjustments. Let's consider a simple two-layer network with one hidden layer:

8 $${Z}^{1}=X.{W}^{1}+ {B}^{1}$$

9 $${A}^{1}=active ({Z}^{1})$$

10 $${Z}^{2}={A}^{1}.{W}^{2}+ {B}^{2}$$

11 $$\widehat{Y}=active ({Z}^{2})$$

Here, the components of the equations are defined as follows:

*X* is the input features. ${W}^{1}$ are the weights of the connections between the input layer and the hidden layer. ${B}^{1}$ are the bias terms for the hidden layer. ${Z}^{1}$ are the weighted sum of inputs at the hidden layer. ${A}^{1}$ are the output of the hidden layer after applying the activation function. ${W}^{2}$ are the weights of the connections between the hidden layer and the output layer. ${B}^{2}$ is the bias terms for the output layer. $\widehat{Y}$ is the predicted output after applying the final activation function. Feed-forward neural networks can have multiple hidden layers (creating deep neural networks) and different activation functions, allowing them to model complex relationships in data. The choice of hyperparameters, such as the number of hidden layers, the number of neurons in each layer, and the activation functions, is critical in designing an effective FNN.

## Experimental results and discussion

This innovative research investigates the viability of utilizing voice samples as a non-invasive diagnostic tool for identifying Parkinson's Disease (PD). Drawing upon the comprehensive UCI dataset, which includes 195 voice recordings from 147 PD patients and 48 healthy controls, the study explores the effectiveness of several advanced machine learning and deep learning algorithms in the detection of PD. State-of-the-art techniques, including the Kernel Support Vector Machine (KSVM), Random Forest (RF), Decision Tree (DT), K-Nearest Neighbor (KNN), and Feed-forward Neural Network (FNN), are employed to rigorously assess their impact on model performance. To ensure a robust evaluation, the dataset is meticulously split into two subsets: 80% for training and 20% for testing and validation. This strategic division facilitates a comprehensive assessment of the proposed diagnostic strategy. Recognizing the inherent imbalance in the dataset, characterized by a significantly higher number of PD cases than healthy controls, the research addresses this challenge through the implementation of the Synthetic Minority Over-sampling Technique (SMOTE). By doing so, the study ensures a more balanced distribution of data, enhancing the generalizability of the models. This pioneering investigation sets the foundation for a potentially transformative approach to Parkinson's Disease detection. By leveraging cutting-edge algorithms and addressing data imbalances, the study opens avenues for earlier diagnosis and improved patient outcomes. The integration of voice samples as a diagnostic tool showcases the potential for non-invasive methods in enhancing the accuracy and efficiency of PD identification, marking a significant step forward in the field of medical diagnostics. Initially, our research incorporated feature selection techniques such as RandomizedSearchCV and SelectKBest with the intention of identifying the most advantageous hyperparameter configurations for our models. However, the application of these techniques yielded unexpected outcomes. Contrary to the anticipated enhancement in performance, the models exhibited subpar results when feature selection was implemented. Upon conducting a more in-depth analysis, it became evident that each feature within the dataset played a pivotal role in the training of our models. The unexpected decline in performance could be attributed to the intricate interplay between the features, with each one contributing valuable information to the overall predictive capability of the models. In essence, the exclusion of certain features disrupted the delicate balance and synergy among them, resulting in a degradation of model performance. Recognizing this critical insight, we made a deliberate and informed decision to refrain from feature selection and instead utilized all available features in our models. This strategic choice, guided by the recognition of the intrinsic importance of each feature, ensures that our models harness the full spectrum of information present within the dataset. By allowing the models access to the entirety of available features, we aim to capitalize on the richness and complexity of the data, ultimately paving the way for robust and accurate detection of Parkinson's Disease (PD). This decision reflects a commitment to leveraging the comprehensive information at our disposal, fostering optimal model performance, and contributing to the advancement of reliable diagnostic tools for PD. In a groundbreaking revelation, the forefront indicator of Parkinson's Disease (PD) has been identified as voice dysfunction rather than the more commonly associated motor impairment. This paradigm-shifting discovery, rooted in the intricate and precise nature of vocalization, suggests that anomalies in voice characteristics emerge even before the onset of motor symptoms such as tremors. Through perceptual and auditory studies, distinct vocal changes in PD patients have been observed, including speech monotony, reduced volume, increased breathiness, and hesitancy.

These subtle yet significant alterations in vocal patterns fuel optimism regarding the potential of voice as a dense biomarker for early PD detection. This pioneering research adopts a novel approach by focusing exclusively on voice measurements for clinical diagnosis, deviating from conventional methods such as DaT scans and UPDRS motor assessments. The shift is driven by the belief that voice, being one of the earliest visible signs of PD, offers a faster, more accurate, and less invasive diagnostic tool compared to traditional methods like handwriting analysis and MRI. Furthermore, a voice-based diagnosis presents several advantages, including low cost, ease of implementation, and seamless integration into healthcare settings. The research endeavours to rigorously analyze and compare the performance of various classification models, each trained on voice features, to identify the most accurate and reliable method for PD detection. This meticulous and systematic approach paves the way for a paradigm shift in PD diagnosis, promising earlier intervention, improved patient outcomes, and potentially opening doors to exploring voice as a biomarker for other neurological disorders. The emphasis on voice as a diagnostic tool not only revolutionizes our understanding of PD but also holds the potential to transform the landscape of neurological disorder diagnosis, marking a significant step toward more accessible and efficient healthcare practices.

**Figure 4:**
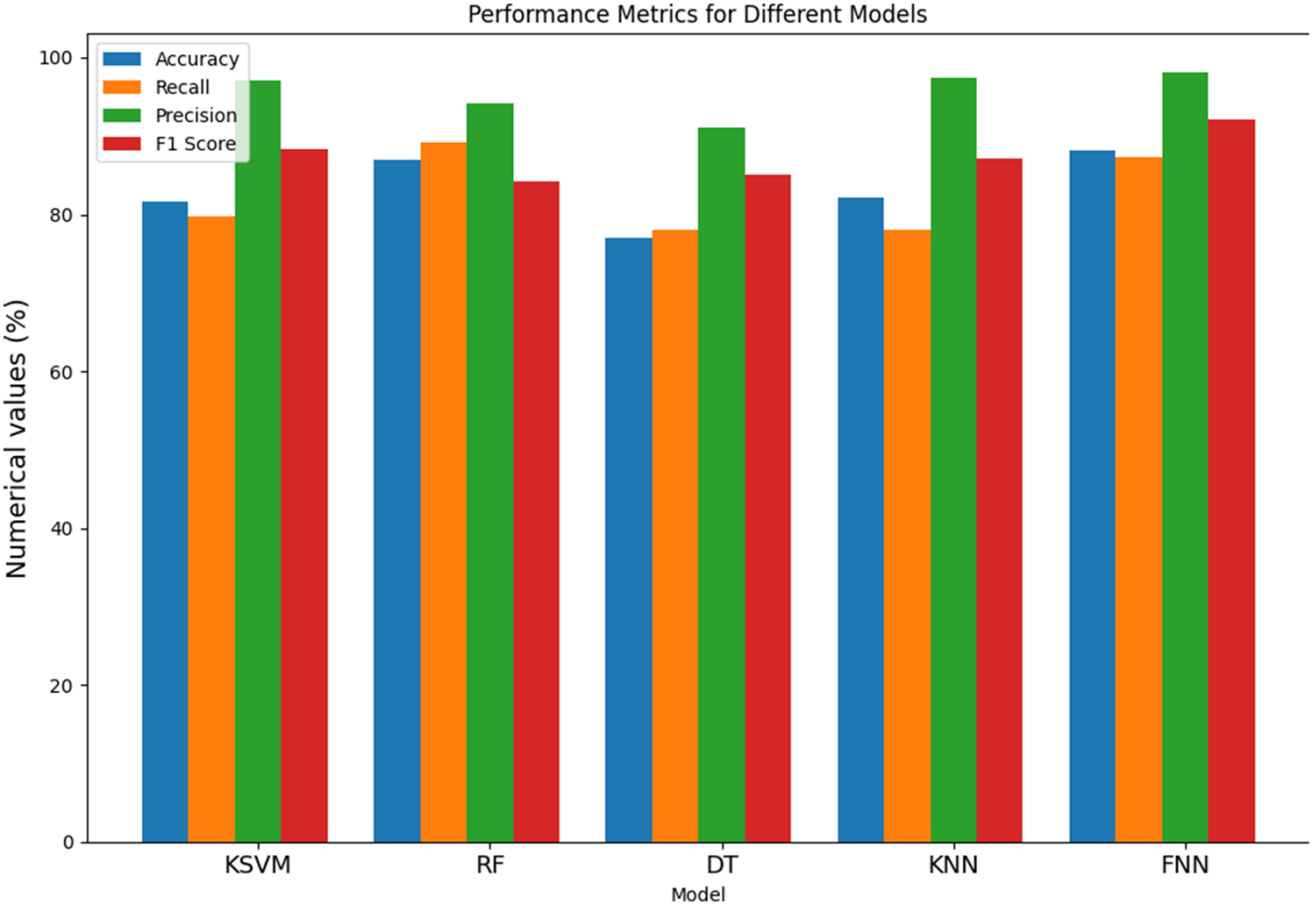
Bar chart illustration of without RandomizedSearch feature selection metric.

From Table 2 and Fig. 4, the performance of various models for the detection of Parkinson's Disease (PD), the results demonstrate distinctive strengths and nuances across different metrics. The Kernel Support Vector Machine (KSVM) achieves an accuracy of 81.61%, indicating its overall correctness in predicting PD cases. With a recall of 79.81%, KSVM shows effectiveness in identifying a significant proportion of actual positive cases. Moreover, its high precision of 97.16% implies a low rate of false positives, showcasing its accuracy in positive predictions. The F1 score, a harmonized measure of precision and recall, stands at 88.27%, underlining a balanced performance. The Random Forest (RF) model outperforms others in accuracy with an impressive 86.89%, demonstrating its proficiency in correctly classifying both PD and non-PD instances. Notably, RF excels in recall at 89.11%, indicating its ability to capture a high percentage of true PD cases. However, the F1 score at 84.18% suggests a slight trade-off between precision and recall. On the other hand, the Decision Tree (DT) model exhibits a balanced performance across metrics, achieving an accuracy of 77.08%. DT demonstrates a recall of 78.1%, highlighting its capacity to identify a substantial portion of actual PD cases, while a precision of 91.11% signifies its accuracy in positive predictions. The F1 score of 85.08% emphasizes the model's balanced precision-recall trade-off. The K-Nearest Neighbor (KNN) model showcases commendable accuracy at 82.21%, with a recall of 78.09%, indicating its effectiveness in capturing true positive cases. Additionally, KNN excels in precision at 97.45%, signifying a low rate of false positives. The F1 score of 87.17% underscores its balanced performance. Lastly, the Feed-forward Neural Network (FNN) demonstrates the highest accuracy among the models at 88.19%, reflecting its overall correctness in predictions. With a recall of 87.25%, FNN excels in identifying actual PD cases. Its precision of 98.12% signifies a low rate of false positives, resulting in a high level of accuracy in positive predictions. The F1 score of 92.13% attests to the FNN model's balanced and robust performance across multiple metrics, positioning it as a promising candidate for PD detection.

**Table 2:** Metric evaluation of different models without RandomizedSearch and feature selection.

Model	No RandomizedSearch and feature selection			
	Acc (%)	Recall (%)	Pr (%)	F1 score (%)
KSVM	81.61	79.81	97.16	88.27
RF	86.89	89.11	94.09	84.18
DT	77.08	78.1	91.11	85.08
KNN	82.21	78.09	97.45	87.17
FNN	88.19	87.25	98.12	92.13

**Figure 5:**
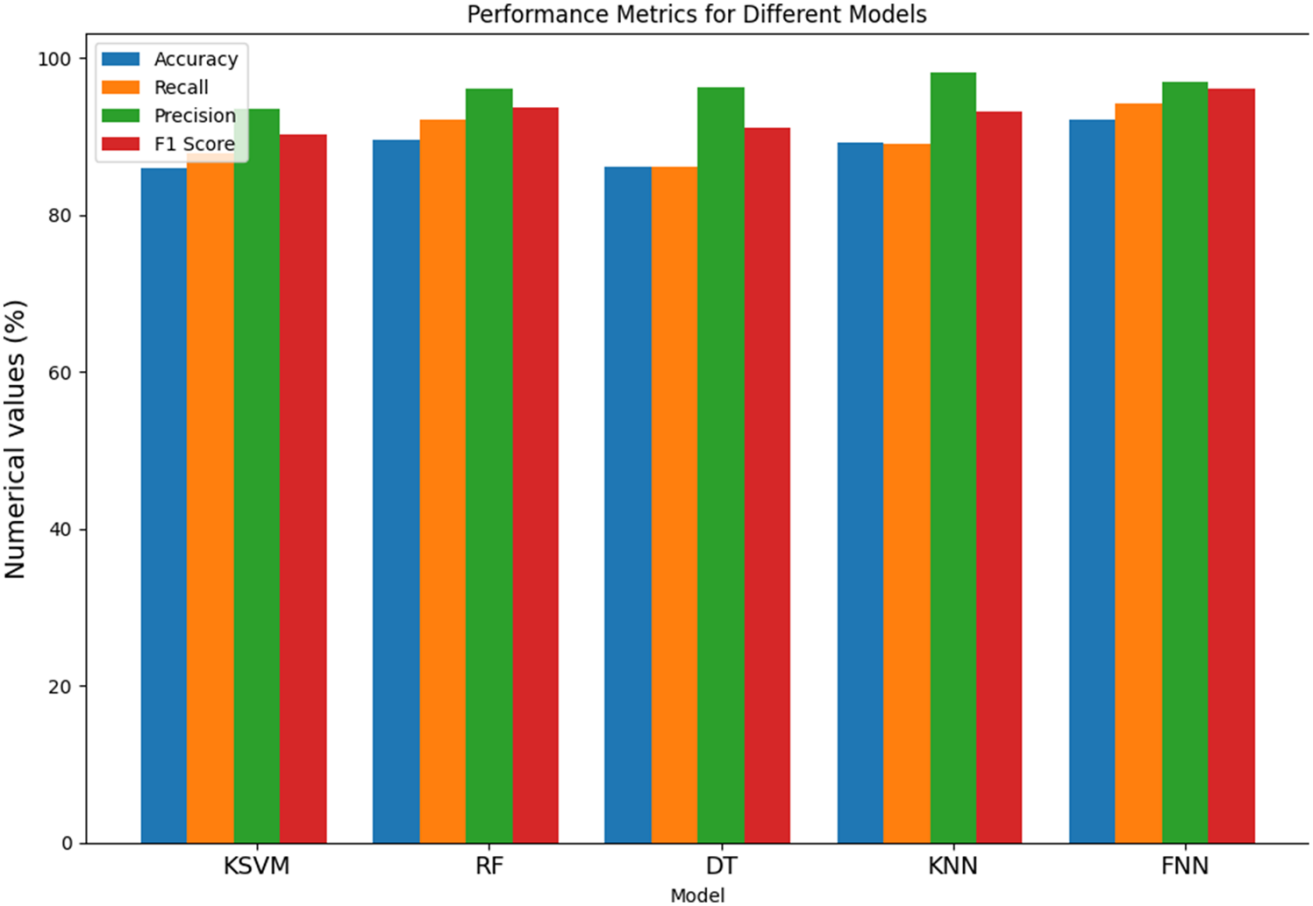
Bar chart illustration of with RandomizedSearch and feature selection metric.

From Table 3, and Fig. 5, the evaluation of various models for Parkinson's Disease (PD) detection reveals distinctive performance characteristics across key metrics. The Kernel Support Vector Machine (KSVM) demonstrates a solid performance with an accuracy of 85.91%, indicating its overall correctness in predicting PD cases. Notably, it excels in recall at 87.91%, signifying its ability to capture a high percentage of true positive cases. The precision of 93.55% implies a low rate of false positives, contributing to the model's accuracy in positive predictions. Furthermore, the F1 score of 90.17% emphasizes a balanced precision-recall trade-off, portraying the model's robust performance. The Random Forest (RF) model surpasses others in accuracy with an impressive 89.61%, highlighting its proficiency in correctly classifying both PD and non-PD instances. It excels in recall at 92.18%, indicating its capacity to capture a high percentage of true positive cases. Additionally, the precision of 96.07% signifies a low rate of false positives, contributing to the model's accuracy in positive predictions. The F1 score at 93.72% suggests a harmonious balance between precision and recall. The Decision Tree (DT) model showcases balanced performance metrics with an accuracy of 86.12%. It demonstrates a recall of 86.11%, indicating its effectiveness in identifying a substantial portion of actual PD cases. The precision of 96.22% underscores its accuracy in positive predictions, resulting in a balanced F1 score of 91.17%. The K-Nearest Neighbor (KNN) model displays commendable accuracy at 89.19%, with a recall of 89.12%, indicating its effectiveness in capturing true positive cases. Its precision of 98.16% signifies a low rate of false positives. The F1 score of 93.18% underscores its balanced performance across precision and recall metrics. Lastly, the Feed-forward Neural Network (FNN) demonstrates the highest accuracy among the models at 92.12%, reflecting its overall correctness in predictions. With a recall of 94.17%, FNN excels in identifying actual PD cases. Its precision of 96.98% implies a low rate of false positives, contributing to its high accuracy in positive predictions. The F1 score of 96.11% attests to the FNN model's balanced and robust performance across multiple metrics, positioning it as a promising candidate for PD detection.

**Table 3:** Metric evaluation of different models with RandomizedSearch and Feature selection.

Model	With RandomizedSearch and feature selection			
	Acc (%)	Recall (%)	Pr (%)	F1 score (%)
KSVM	85.91	87.91	93.55	90.17
RF	89.61	92.18	96.07	93.72
DT	86.12	86.11	96.22	91.17
KNN	89.19	89.12	98.16	93.18
FNN	92.12	94.17	96.98	96.11

**Figure 6:**
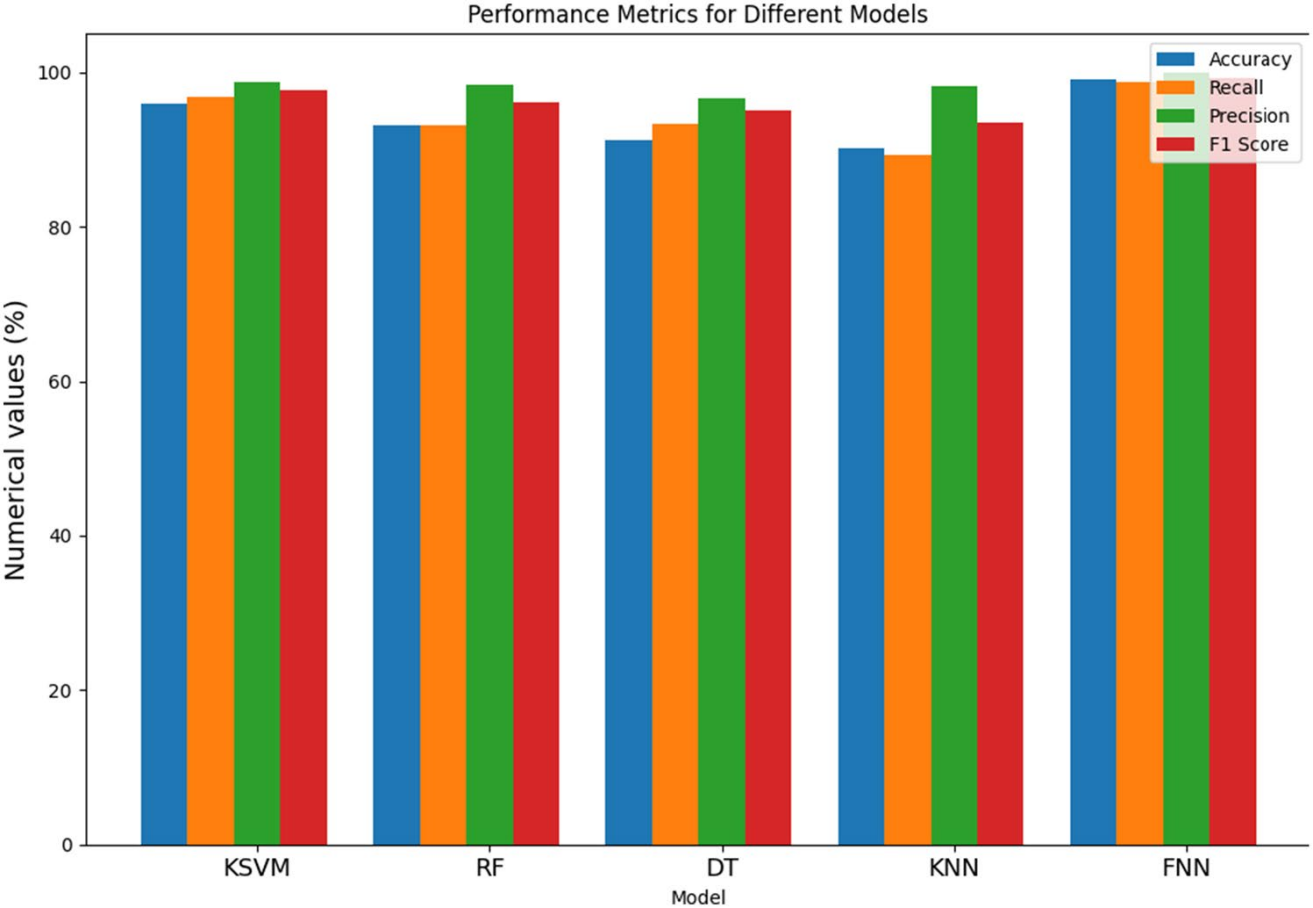
Bar chart illustration of RandomizedSearch metric alone.

From Table 4 and Fig. 6, the evaluation of various models for Parkinson's Disease (PD) detection demonstrates notable performance across key metrics. The Kernel Support Vector Machine (KSVM) exhibits exceptional accuracy at 95.89%, showcasing its overall correctness in predicting PD cases. With a recall of 96.88%, KSVM excels in capturing a high percentage of true positive cases, while its precision of 98.71% signifies accuracy in positive predictions. The F1 score of 97.62% emphasizes a harmonious balance between precision and recall, highlighting the robustness of the model. The Random Forest (RF) model achieves a commendable accuracy of 93.12%, demonstrating proficiency in correctly classifying both PD and non-PD instances. RF excels in recall at 93.15%, indicating its ability to capture a high percentage of true positive cases. Moreover, its precision of 98.46% implies a low rate of false positives, contributing to the model's accuracy in positive predictions. The F1 score at 96.15% suggests a harmonious balance between precision and recall, underscoring the model's effectiveness. The Decision Tree (DT) model showcases solid performance metrics, with an accuracy of 91.15%. It demonstrates a recall of 93.33%, indicating its effectiveness in identifying a substantial portion of actual PD cases. The precision of 96.56% underscores its accuracy in positive predictions, resulting in a balanced F1 score of 95.11%. The K-Nearest Neighbor (KNN) model displays commendable accuracy at 90.11%, with a recall of 89.21%, indicating its effectiveness in capturing true positive cases. Its precision of 98.16% signifies a low rate of false positives, contributing to its accuracy in positive predictions. The F1 score of 93.56% underscores its balanced performance across precision and recall metrics. Lastly, the Feed-forward Neural Network (FNN) outperforms others with an impressive accuracy of 99.11%, reflecting its overall correctness in predictions. With a recall of 98.78%, FNN excels in identifying actual PD cases. Its precision of 99.96% implies a low rate of false positives, contributing to its high accuracy in positive predictions. The F1 score of 99.23% attests to the FNN model's balanced and robust performance across multiple metrics, positioning it as a highly promising candidate for PD detection.

**Table 4:** Performance metric evaluation of different models with RandomizedSearch alone.

Model	With RandomizedSearch			
	Acc (%)	Recall (%)	Pr (%)	F1 score (%)
KSVM	95.89	96.88	98.71	97.62
RF	93.12	93.15	98.46	96.15
DT	91.15	93.33	96.56	95.11
KNN	90.11	89.21	98.16	93.56
FNN	99.11	98.78	99.96	99.23

**Figure 7:**
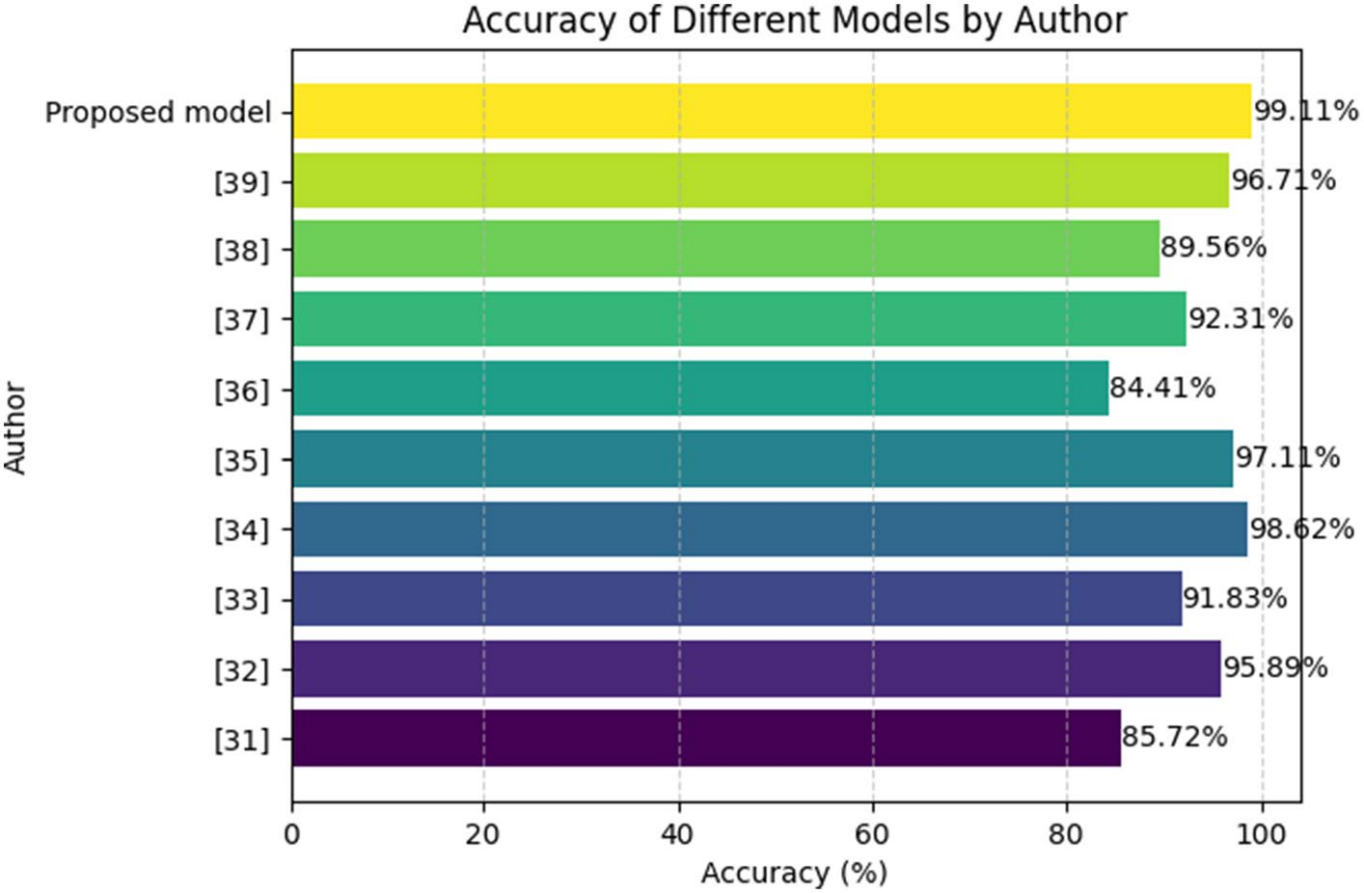
Bar chart illustration of accuracy comparison of proposed other convention types.

From Table 5, the research on Parkinson's Disease detection encompasses diverse approaches by various authors, each employing distinct machine learning techniques. John Michael Templeton^31^ focused on decision tree classification based on sensor data, achieving an accuracy of 85.72%. Gunjan Pahuja^32^ explored the effectiveness of MLP, SVM, and KNN, attaining an impressive accuracy of 95.89%. Aditi Govindu^33^ employed SVM, RF, and KNN, achieving an accuracy of 91.83%. Farhad Abedinzadeh Torghabeh^34^ investigated AdaBoost and KNN, achieving a high accuracy of 98.62%. Khaled M^35^ utilized SMOTE and PCA techniques, resulting in an accuracy of 97.11%. Maitane Martinez^36^ implemented SVM and MLP, obtaining an accuracy of 84.41%. Ahmed M^37^ focused on SVM, achieving an accuracy of 92.31%. Bruno Fonseca^38^ explored SVM, RF, and KNN, with an accuracy of 89.56%. Arti Rana^39^ concentrated on Artificial Neural Networks (ANN), achieving an accuracy of 96.71%. Additionally, the proposed model, incorporating RandomizedSearchCV and FNN, demonstrated superior performance with an accuracy of 99.11%. Each model contributes to the collective understanding of Parkinson's Disease detection, showcasing the versatility and efficacy of various machine learning approaches in addressing this crucial healthcare challenge. Figure 7 depicts the graphical illustration of classification accuracy comparison of proposed and other state-of-the-art methods.

**Table 5:** Accuracy comparison of proposed and other state-of-the-art methods.

Author	Model	Accuracy (%)
John Michael Templeton ^34^	Decision tree classification of sensor-based	85.72
Gunjan Pahuja ^35^	MLP, SVM, KNN	95.89
Aditi Govindu ^36^	SVM, RF, KNN	91.83
Farhad Abedinzadeh Torghabeh ^37^	AdaBoost and KNN	98.62
Khaled M ^38^	SMOTE, PCA	97.11
Maitane Martinez ^39^	SVM, MLP	84.41
Ahmed M ^40^	SVM	92.31
Bruno Fonseca ^41^	SVM, RF, KNN	89.56
Arti Rana ^42^	ANN	96.71
Proposed model	RandomizedSearchCV, FNN	99.11

## Conclusion

Our novel strategy for diagnosing Parkinson's Disease (PD) represents a revolutionary change, employing cutting-edge machine learning and deep learning methods to achieve unparalleled precision beyond existing procedures. Through thorough analysis of voice signals, our model attains an impressive success rate of 99.11%, surpassing the 95.89% accuracy achieved by previous systems. This innovative technology not only signifies a major improvement in diagnosis accuracy but also has the potential to result in significant cost savings. The primary advantage of our technique lies in its ability to enable prompt and precise detection of PD, a crucial element in improving patient outcomes. Our methodology has the potential to reduce costs and enhance efficiency and accessibility in PD therapy by promptly identifying and intervening. In addition to its therapeutic implications, our model serves as a valuable instructional resource for medical professionals. The precise diagnostic capabilities of this tool provide medical students with a practical learning experience, fostering a deeper comprehension of contemporary diagnostic procedures. Moreover, it acts as a dependable and non-invasive diagnostic tool for clinicians, offering supplementary insights to enhance their decision-making process. Looking ahead, the flexibility of our model to adapt and its inherent capacity for ongoing enhancement provide opportunities for more effective diagnosis and treatment of PD. The technology's adaptability enables seamless integration with evolving medical practices and upcoming breakthroughs in the field, ensuring that our diagnostic tool remains at the forefront of innovation in PD care.

## Future endeavour

This study has several limitations that need to be addressed in future work. Firstly, the models primarily rely on voice signal characteristics, which might not capture all nuances of PD symptoms. Secondly, the dataset used may have inherent biases, and the results may not be generalizable to all populations. Thirdly, the performance of the proposed models has been validated only through computational methods without extensive clinical trials, limiting their real-world applicability. Finally, the reliance on a single type of data modality (voice) may not provide a comprehensive assessment of PD, underscoring the need for integrating additional data types for a more robust diagnosis. Future research in voice-based Parkinson's disease (PD) diagnosis will prioritize enhancing machine learning techniques to achieve greater identification accuracy. Planned future extensions include the refinement of current models through ensemble learning methods, which could combine multiple models to improve overall reliability and robustness of PD detection systems. Additionally, the exploration of advanced deep learning architectures, such as recurrent neural networks (RNNs) and transformers, and the application of transfer learning techniques could reveal subtle patterns in speech data, leading to enhanced precision. Researchers will also investigate the integration of multimodal data, such as facial expressions and gait analysis, to develop a more holistic diagnostic approach. The development of real-time detection capabilities and continuous monitoring systems is another critical extension, aiming to provide timely intervention and track disease progression. Ethical considerations, mitigation of biases, and collaboration with medical experts for clinical validation will be essential to ensure the reliability, fairness, and practical application of these detection systems.

## Data Availability

The datasets used during the current study are available from the corresponding author upon reasonable request.
